# Agreement of Mammographic Measures of Volumetric Breast Density to MRI

**DOI:** 10.1371/journal.pone.0081653

**Published:** 2013-12-04

**Authors:** Jeff Wang, Ania Azziz, Bo Fan, Serghei Malkov, Catherine Klifa, David Newitt, Silaja Yitta, Nola Hylton, Karla Kerlikowske, John A. Shepherd

**Affiliations:** 1 Department of Radiology and Biomedical Imaging, University of California San Francisco, San Francisco, California, United States of America; 2 Synarc, Inc., Newark, California, United States of America; 3 Departments of Medicine and Epidemiology/Biostatistics, University of California San Francisco, San Francisco, California, United States of America; Kyushu University Faculty of Medical Science, Japan

## Abstract

**Background:**

Clinical scores of mammographic breast density are highly subjective. Automated technologies for mammography exist to quantify breast density objectively, but the technique that most accurately measures the quantity of breast fibroglandular tissue is not known.

**Purpose:**

To compare the agreement of three automated mammographic techniques for measuring volumetric breast density with a quantitative volumetric MRI-based technique in a screening population.

**Materials and Methods:**

Women were selected from the UCSF Medical Center screening population that had received both a screening MRI and digital mammogram within one year of each other, had Breast Imaging Reporting and Data System (BI-RADS) assessments of normal or benign finding, and no history of breast cancer or surgery. Agreement was assessed of three mammographic techniques (Single-energy X-ray Absorptiometry [SXA], Quantra, and Volpara) with MRI for percent fibroglandular tissue volume, absolute fibroglandular tissue volume, and total breast volume.

**Results:**

Among 99 women, the automated mammographic density techniques were correlated with MRI measures with R^2^ values ranging from 0.40 (log fibroglandular volume) to 0.91 (total breast volume). Substantial agreement measured by kappa statistic was found between all percent fibroglandular tissue measures (0.72 to 0.63), but only moderate agreement for log fibroglandular volumes. The kappa statistics for all percent density measures were highest in the comparisons of the SXA and MRI results. The largest error source between MRI and the mammography techniques was found to be differences in measures of total breast volume.

**Conclusion:**

Automated volumetric fibroglandular tissue measures from screening digital mammograms were in substantial agreement with MRI and if associated with breast cancer could be used in clinical practice to enhance risk assessment and prevention.

## Introduction

Other than age and specific genetic mutations, dense breast tissue is one of the strongest risk factors to predict who will develop breast cancer [1], [2]. Like body mass index, it is one of the few modifiable breast cancer risk factors. Breast density also impacts the sensitivity of mammography. Sensitivity decreases from over 90% in women with low breast density to less than 60% for women with high breast density [3], [4]. In addition, monitoring for a density reduction in women taking risk reduction therapy can in some cases be an effective method to monitor whether a woman is responding to therapy [5], [6], [7], [8], [9], [10]. Several states in the United States, including California, Texas, and Connecticut, have recognized the influence of breast density on cancer detection and require the reporting of breast density to women with dense breasts as part of their mammography examination. However, how to report breast density is under debate.

Mammographic breast density in clinical practice is assessed using a 4-category score defined in the American College of Radiology's Breast Imaging Reporting and Data System (BI-RADS) [11]. Several risk models have been developed using BI-RADS density including the Breast Cancer Surveillance Consortium's 1-year and 5-year models, developed using over 1 million women [12], [13]. BI-RADS density categories, however, have limitations. Agreement between radiologists is only moderate, with poor agreement between radiologists in the middle two density categories [14]. Second, BI-RADS categories are too coarse to monitor breast density changes in individual women on prevention therapy: approximately 6–9% over 2 years for Tamoxifen [5], [10], [15].

Continuous and objective measures of breast density, such as percentage areal [16] and volumetric [17] mammographic density, were developed to improve on the semi-quantitative and subjective nature of scoring. Percent areal density has been shown to have a slightly stronger risk association with breast cancer than categorical scores [18], but does not represent the true volume of dense tissue, could have errors associated with its two-dimensional projection, and requires a trained reader [19].

*In vivo* tissue volume and mass measures can be made from projection x-ray images. One example measures fat and lean mass using dual-energy x-ray absorptiometry [20], [21]. Several mammographic measures of absolute volumetric breast density have also been reported [22], [23], [24], [25], but agreement between different methods of quantifying breast density is unclear. Three-dimensional imaging methods, such as MRI or CT, can be used to examine agreement with volumetric measures derived from projection x-ray systems. In 3D MRI and CT images, adipose and fibroglandular tissues do not overlap because the organ is imaged from several angles and reconstructed in 3-dimensions. Such images can therefore be easily segmented to quantify breast tissue compartments volumetrically [26], [27].

In this study, we compared the agreement of three measures of automatic volumetric mammographic breast density to MRI breast density to determine the accuracy of volumetric breast density from mammography.

## Methods

The study design was a retrospective analysis to compare breast density measured from screening MRI exams to that from screening mammograms on a population of women referred for both. Four methods were used to assess volumetric breast density: a fuzzy-clustering segmentation method on MRI [28], the Single-energy X-ray Absorptiometry (SXA) method [29], the Quantra method (Hologic, Inc., Bedford, MA, USA), and the Volpara method (Mātakina, Wellington, New Zealand) on Full-Field Digital Mammography (FFDM) images [30]. The study was fully HIPAA compliant and approved by the University of California, San Francisco (UCSF) Institutional Review Board for passive consenting processes or a waiver of consent to enroll participants, link data, and perform analyses for research purposes. A Federal Certificate of Confidentiality also protects the identities of research subjects.

### Subjects

Women aged 18 years or older undergoing screening mammography and screening MRI between 2007 and 2010 at UCSF were included. Sample size was not estimated before beginning the study. For a woman to have been included in the study, she must have had a set of screening digital mammograms and a screening MRI exam acquired within 1 year of each other, have completed a breast health questionnaire, had no previous history of breast cancer or breast surgery, and have had a BI-RADS assessment of either 1 or 2 (negative or benign finding, respectively). Only images of the left laterality were used for the entire study, of craniocaudal (LCC) views for mammography. Each subject contributed one LCC mammogram matched to a left breast MRI. When multiple mammography examinations were available within the study period, that closest in date to the MRI exam was used.

### MR imaging and breast density analysis

T1-weighted non-contrast fat-saturated images were acquired on either a 1.5 or 3 Tesla GE system (General Electric Medical Systems, Milwaukee, WI) using a bilateral phased-array breast coil (Medical Devices, Madison, WI) with women lying in a prone position. Slice thicknesses across subject were consistently 2 mm, though in-plane spatial resolutions varied with breast size, averaging approximately 0.7 mm×0.7 mm. The images were analyzed using a quantitative fuzzy C-means (FCM) technique previously described [28]. Percent fibroglandular volume (%FGV) was calculated as the ratio of clusters of high intensity voxels determined to be fibroglandular volume (FGV) to total breast volume (TBV). The reader reviewed all slices to insure accuracy of the segmentation of fibroglandular tissue.

### Mammographic imaging

All mammograms were acquired on one of six Hologic Selenia FFDM systems at UCSF. These systems used a molybdenum anode x-ray tube and have a pixel spatial resolution of 70 µm×70 µm. The raw (“For Processing”) format images were archived, from which all analyses for mammographic techniques were obtained. The images available to the study were collected as part of a large cohort study and previously downsized by 50% in both dimensions before analysis to conserve server storage space, creating 140 µm×140 µm averaged pixels. These downsized images were used directly for SXA analysis and upsized to original dimensions for Quantra and Volpara analyses. A test sample of images were compared using all three mammographic density techniques before and after downsizing and no significant differences were found in breast volume, FGV, and %FGV measures.

### SXA breast density analysis

SXA is an established method for measuring breast density, which previously has been described [17], [29]. In brief, the technique compares the breast image pixel grayscale values to that of a reference phantom that is imaged with the breast. Additional weekly quality control scans were acquired using a phantom named GEN III. GEN III was imaged in the location normally occupied by a breast and was constructed with three tissue-equivalent density materials at three thicknesses as well as other features to test the mammography system's geometric accuracy. Differences between the SXA calibration and the GEN III measures were used to update the SXA calibration continually. This study reports results using version 7.1 of the SXA analysis software package (UCSF, San Francisco, CA).

### Quantra and Volpara volumetric assessment

Quantra and Volpara are FDA approved, commercially available, and fully-automated software for estimating volumetric breast density. Quantra has been previously described by Harman et al [30] and Volpara by Aiken et al [31]. Version 3.2 of the Quantra Algorithm (Cenova 1.3) and version 1.4.3 of the Volpara Algorithm (Imaging Software 1.5.7) were used in this study. In both, FGV is found by referencing each pixel's attenuation to the attenuation of pixels that are labeled as exclusively adipose (i.e. the lowest attenuation pixels). The estimated FGV is then divided by the TBV to calculate the %FGV of the breast.

There are three primary differences between the two commercial mammographic methods (Quantra and Volpara), FCM technique for MRI, and SXA algorithms regarding how they define FGV and %FGV:

1. Reference Definition: Quantra, Volpara, and the FCM use relative references of adipose tissue attenuation (Quantra, Volpara) or signal intensity (FCM), defining the area with the lowest value in each woman's image as pure adipose, while SXA uses an in-image phantom with fixed fibroglandular density and fat references. In addition, SXA defines the total lack of dense breast tissue (i.e. 0%FGV) as pure fat (versus adipose, containing both fat and water). If the fractions of fat and water in adipose tissue were the same in all women, then SXA would still differ from the FCM and the commercial systems' calibrations due to the water volume in adipose. The SXA algorithm does this because phantom references for breast adipose have been found to be too dense [32], [33] and because a fixed reference is always available even in dense breasts without many available pixels of pure adipose tissue.
2. Tissue Compartment Model: Quantra and Volpara use a 3-compartment model of skin, fibroglandular, and adipose tissue [30], while SXA and FCM utilize 2-compartment models; fat and fibroglandular for SXA and fat and water for FCM.
3. Pixel/Voxel Subdivision: SXA, Quantra, and Volpara make no assumptions with regards to labeling pixels as either all adipose or fibroglandular, but ultimately subdivide each pixel into some fraction of fibroglandular and a second compartment (fat for SXA and adipose+skin for Quantra and Volpara). In contrast FCM groups each voxel to either a fat or fibroglandular tissue cluster.

A modified SXA model, SXA without adipose water volume, was derived to assess compatibility of the MRI and SXA models further by approximating the hydration of adipose to be 15% water. Adipose volume was defined as TBV less the FGV. Previous work using water saturated MRI has estimated the adipose water volume as 8% [34] to 20% [35] of the total adipose volume. After normalizing for any differences in measured TBV between the MRI and SXA, the adipose water volume was subtracted from the SXA FGV.

### Statistics

Descriptive statistics of the subjects' age, weight, and BMI were used to summarize the population. The frequencies of first degree family history of breast cancer and BI-RADS density categorization were also calculated. Descriptive statistics of the mammographic and MRI density measures were used to summarize the breast density data. Medians and inter-quartile ranges were used instead of means and standard deviations with the density measures, as their distributions were not normal. Wilcoxon signed-rank tests were used to determine whether mammographic measures' were significantly different than those of MRI.

Linear regressions were performed to model the relationships between mammographic and MRI breast density measures of FGV, log FGV, and %FGV, using difference in time between exams as a possible covariate. The natural logarithm of FGV was used to normalize its distribution and to be comparable to our previous reporting of these measures, where the log transformation was found amongst a variety of scaling factors to best normalize and improve breast cancer risk classification ability of the FGV measure [17]. Squared Pearson's correlation coefficients were calculated from the relationships of density measures between methods. Differences between regression equation fit parameters were tested with paired Student's t-tests for significance. Root-mean-square errors (RMSE) were also calculated to aggregate the magnitude of individual differences between mammographically and MRI derived measures.

Quartile groupings based on distributions of %FGV and log FGV measures were compared using weighted kappa statistics to determine clinical agreement between measures. We interpreted the kappa statistics with the following categories [36]: slight agreement (less than or equal to 0.20), fair agreement (0.21 to 0.40), moderate agreement (0.41 to 0.60), substantial agreement (0.61 to 0.80), and almost perfect agreement (0.81 to 1.00).

All statistical analyses were performed using SAS Version 9.3 software (SAS Institute, Cary, NC) and p-values of less than 0.05 interpreted as significant.

## Results

**Figure 1:**
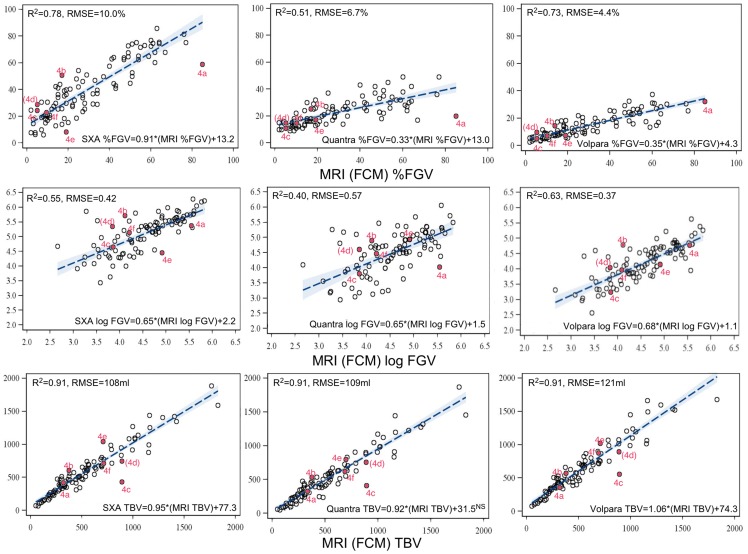
Best linear regression fit line with 95% confidence interval bands for percentage fibroglandular density (top), log fibroglandular volume (middle), and total breast volume (bottom) for MRI versus either SXA (left), Quantra (center), or Volpara (right) measures. Solid points correspond to example images in Figure 4.

Ninety-nine women met selection criteria. A high proportion of women had a family history of breast cancer and most had normal BMI (Table 1). All MRI exams were within 1 year of the mammography visit. The median breast density and quartile ranges are shown for each density measure in Table 2 and Table 3 respectively. Linear regression plots with regression coefficients comparing the mammography techniques to MRI for %FGV, log FGV, and TBV are shown in Figure 1. All mammographic measures of TBV were highly correlated to MRI TBV (R^2^ = 0.91 for all three). Volpara showed a higher correlation to MRI for log FGV while SXA showed the highest correlation to MRI for %FGV. All best fit regression lines had significant intercepts (mammographic value not 0 when MRI value is 0), except that of Quantra vs MRI TBV. In general, the mammographically-derived values tracked MRI values in a similar way for log FGV and TBV (SXA to MRI slopes of 0.65, and 0.95, Quantra to MRI slopes of 0.65 and 0.92, and Volpara to MRI slopes of 0.68 and 1.06 respectively). However, the slopes were substantially different for %FGV to MRI (0.91, 0.33 and 0.35 for SXA, Quantra, and Volpara respectively). The time difference between the mammogram and MRI acquisition was not significantly associated with any measures except %FGV for SXA and FGV for Quantra. Including time between MRI and mammography measures explained less than 3% of the variance (not shown). RMSE results are also shown in Figure 1, ranging from 4.4% to 10% for %FGV, 0.37 to 0.42 for FGV, and 108 ml to 121 ml for TBV.

**Table 1:** Description of population characteristics.

Demographic Variables		
Age (years), mean (Std dev) min/max	47.2 (12.1)	26/79
Weight (pounds), mean (Std dev) min/max	139.1 (24.1)	85/260
BMI (kg/m^2^), mean (Std dev) min/max	23.1 (3.9)	14.6/46.1
**Frequency Variables**		
1^st^ degree family history, n %		
No	21	21.2
Yes	78	77.7
BI-RADS density category, n %		
1	12	12.1
2	35	35.4
3	30	30.3
4	22	22.2

BMI = Body Mass Index, Std dev = standard deviation.

**Table 2:** Density measures for study participants by method (n = 99).

Density Measures	Median (IQR)	Min	Max
**Percent Fibroglandular Volume (%)**			
SXA	36.7* (38.8)	6.1	85.8
Quantra	22.0* (14.0)	9.0	49.0
Volpara	13.3* (12.6)	2.6	37.3
MRI	24.0 (36.0)	2.0	85.0
**Absolute Fibroglandular Volume (ml)**			
SXA	192.6* (160.5)	31.1	533.8
Quantra	101.0* (102.0)	9.0	425.0
Volpara	64.8* (61.6)	13.0	278.2
MRI	102.3 (120.4)	14.3	338.1
**Total Breast Volume (ml)**			
SXA	514.0* (383.3)	69.4	1882.8
Quantra	441.0* (379.0)	51.0	1870.0
Volpara	558.3* (452.3)	76.2	2178.3
MRI	460.4 (412.1)	49.9	1828.4

**Table 3:** Percent fibroglandular density quartile ranges by method (n = 99).

	Q1	Q2	Q3	Q4
SXA percent fibroglandular volume (%)	6.1–21.4	21.4–36.7	36.7–60.2	60.2–85.8
Quantra percent fibroglandular volume (%)	9.0–15.0	15.0–22.0	22.0–29.0	29.0–49.0
Volpara percent fibroglandular volume (%)	2.6–7.9	7.9–13.3	13.3–20.5	20.5–37.3
MRI percent fibroglandular volume (%)	2.0–13.0	13.0–24.0	24.0–49.0	49.0–85.0

**Figure 2:**
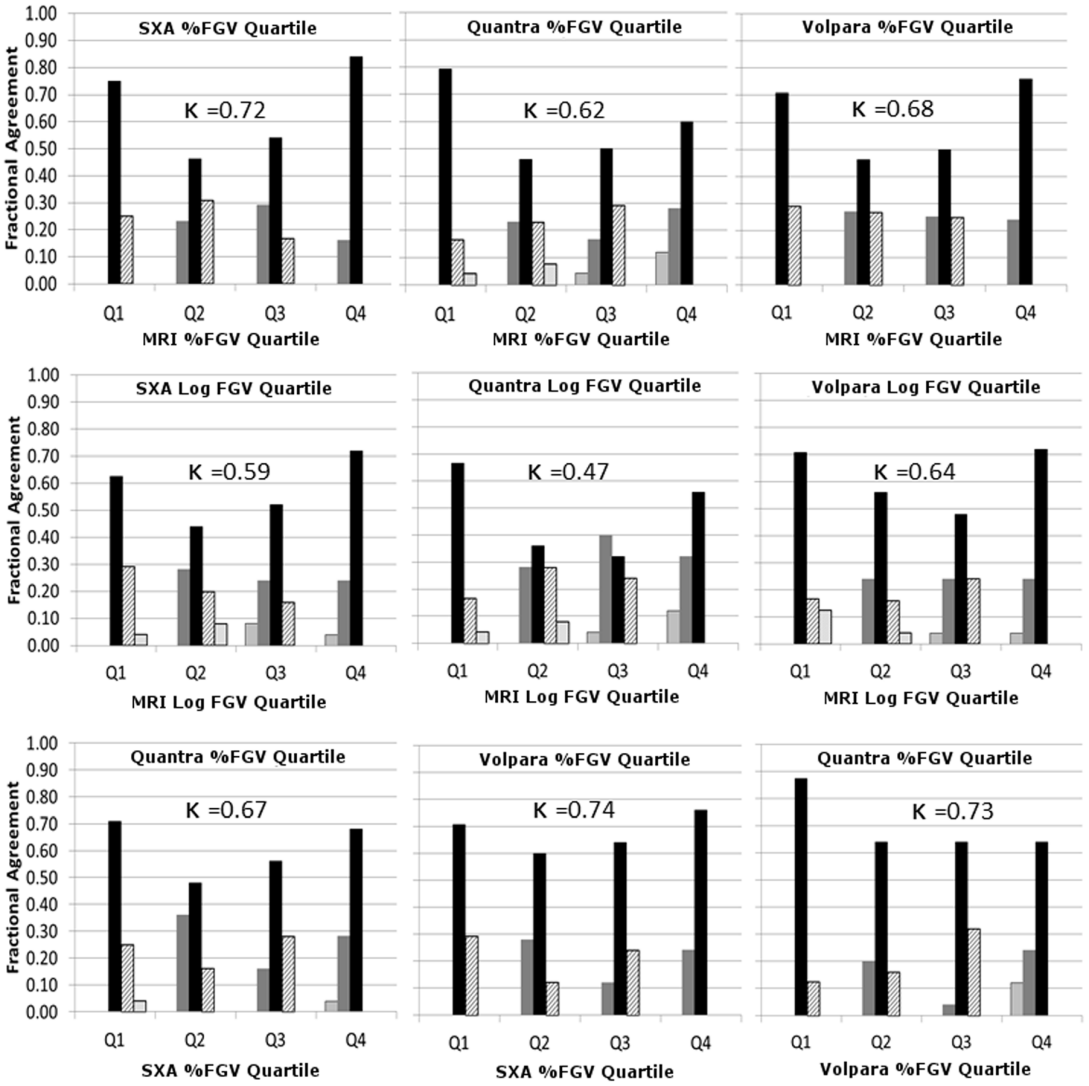
Comparison of quartiles classification for percent fibroglandular density (top) and log fibroglandular volume (middle) for MRI versus SXA (left), Quantra (center), and Volpara (right). The bottom row of plots show quartiles comparisons between mammographic density measures. Legend at right defines categories of agreement, where either the two compared method's agree completely (black) or are off by one or two quartiles up or down in comparison with the other method.

Distribution quartile groups were compared for MRI, SXA, Quantra, and Volpara (Figure 2). Overall, the percentage agreements within each %FGV quartile were similar for each mammographic measure to MRI. SXA %FGV had the highest percentage of agreement to MRI. The mammographic methods placed 60% (Quantra) to 85% (SXA) of the same women in the highest density quartile as MRI. A lower agreement was seen for the middle two quartiles of %FGV, with agreement ranging from 46% to 55%. Similar trends were seen for log FGV. Disagreement of 2 quartiles for density was less common but did exist for Quantra %FGV measures and log FGV for all three mammographic methods. The weighted kappa statistics are also shown in Figure 2 for each relationship in the respective plot. Substantial agreement was found between all density comparisons with the highest kappa coefficient being k = 0.72 for MRI versus %FGV. Moderate agreement was found between all log FGV measures, where weighted kappa scores ranged from k = 0.47 (Quantra vs. MRI) to 0.64 (Volpara vs. MRI). The kappa coefficients for %FGV between mammographic measures was also moderate from 0.67 to 0.74.

**Figure 3:**
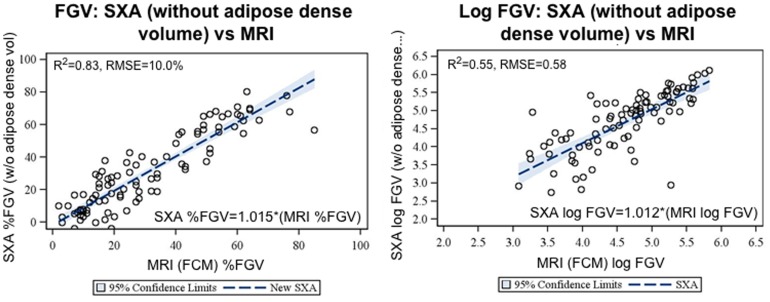
Validation of SXA model using breast biology and adipose volume estimates from MRI. The amount of water volume in the MRI adipose volume was estimated to be 15% of the volume, which is consistent with previous work estimating it to be between 8% [34] and 20% [35]. The MRI model does not include adipose density in the fibroglandular volume while SXA does. Subtracting out the adipose water volume from the SXA fibroglandular volume improved the agreement between SXA and MRI from R^2^ = 0.78 to 0.83 and removed most of the bias between the measures.

In the modified SXA model, where adipose water volume was subtracted from the measures (Figure 3), the best fit slope for log FGV improved from 0.65 to 1.012 and the intercept term decreased from 2.2 to 0.27 (not significant). The best fit slope for SXA %FGV changed from 0.91 to 1.015, and the intercept term decreased from 13.2 to −2.2 (not significant). Agreement between SXA and MRI %FGV improved slightly from κ = 0.72 to 0.74 (not shown).

## Discussion

**Figure 4:**
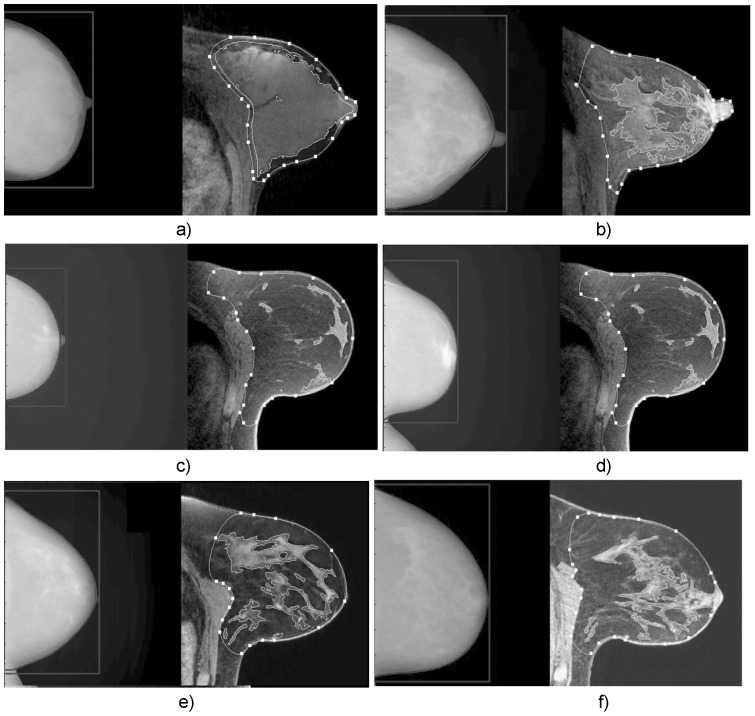
Six comparisons of the LCC mammograms to their respective left central breast axial-slice MR images on five different women (c and d are the same woman). The white line connecting points in the MR images define the total breast volume. The MRI fibroglandular volume is shown delineated with white lines without points. Solid data points 4a–4f in Figure 1 correspond to the image labels a–f. Compared to the mammographically-derived SXA values, a) MRI percent density is higher, b) MRI percent density is lower, c) MRI breast volume is higher, d) of the same woman as c (this mammogram not part of analyses, only here and measures plotted in Figure 1 to illustrate one reason for discrepancy between methods' results), MRI breast volume is better segmented due to the breast being extended more into the mammographic image field, e) MRI breast volume is lower, f) all MRI measures of density and volume were in substantial agreement.

We compared three mammographic techniques to an MRI technique for quantifying volumetric breast density. TBV, as measured by MRI and mammography techniques were well correlated with regression slopes ranging from 0.92 to 1.06 times that of MRI. One may expect that the MRI TBV to be higher because MRI has access to delineate around the pectorals muscle. However, we found that average TBV was higher for SXA and Volpara but lower for Quantra when compared to MRI. We found most cases of disagreement were driven by differences in TBV measured for MRI versus mammography. Figure 4 shows comparative images selected from the results in Figure 1. From Figure 1, the RMSE between SXA and MRI TBV was 108 ml but 65 ml for FGV (not shown), indicating that the observed lack of agreement between mammographic density and MRI density is most likely driven by differences in the total volume. Figure 4a is an example where the difference in TBV caused a large difference in density due to glandular density that extended to the chest wall (i.e. a lack of retroglandular adipose). However, differences seen in Figure 4b seem to be related to the breast having higher attenuation throughout, including the adipose that impacted the mammographic measures. The mammographic images in Figures 4c and 4d were acquired from the same woman on the same day (left, MR images on right are the same) and are examples of how measured TBV can vary substantially due to breast positioning. The mammogram in Figure 4c was a reimage of that in 4d to ensure a good nipple profile in the image. Only that in Figure 4c was used in our study results since we chose the last mammogram acquired for each visit. Figure 4d and its calculated measures plotted in Figure 1 were not actually part of the study's quantitative analyses, but only included to illustrate one reason for discrepancy. Comparing the MR images of Figures 4c to 4e, there were substantial differences in how the TBV was delineated from truncal subcutaneous in the retroglandular region. In Figure 4e the TBV is substantially less than the mammographically-defined measure.

As evidenced in Figure 4, the differences in TBV were mainly due the difficulties in delineating the breast from truncal subcutaneous adipose (for MRI) and variations in breast positioning (for mammography). TBV errors, either by incomplete breast imaging in craniocaudal views or ambiguity in delineating between breast and truncal adipose, seem to be the limiting factor on both accuracy and precision for volumetric breast density. It is unclear if screening mammography mediolateral oblique views would be any better in this regard as this was not tested.

The primary differences between the three mammography techniques and MRI were in the type of references used for defining fibroglandular and adipose tissue. The mammographic techniques do not segregate fibroglandular from adipose tissue while MRI does ultimately label each voxel as one or the other. There were differences in the best fit RMSE values to MRI especially for %FGV and FGV. It was not possible to directly test for explanations. Kallenberg et al. [37] found that paddle tilt correction improved the agreement between both percent and absolute FGV of their mammography measures to MRI. The SXA method also attempts to accurately assess breast thickness variations due to compression paddle tilt and warp, and uses a mammogram-specific phantom for a tissue density reference. The Volpara and Quantra methods are proprietary with respect to corrections they may make regarding paddle tilt. Because the SXA model references to fat and fibroglandular tissue, we expected SXA to have higher %FGV and FGV than MRI, Volpara, or Quantra since the water volume of adipose is included in the SXA FGV compartment. We found that eliminating the adipose water volume from the FGV slightly improved the MRI and SXA agreement.

There are several previous comparisons of MRI to mammographic measures of volumetric breast density. The most methodologically similar study to the present study was smaller (n = 32) and compared Quantra to MRI density, Kontos et al. [38]. The MRI were analyzed using a similar fuzzy C-means segmentation algorithm. Like the present study, TBV and density were found to be highly associated (R^2^ = 0.71 and 0.80 respectively), with the average MRI TBV being higher and density being lower than Quantra. However, Kontos et al. found lower overall association of FGV than in our study (R^2^ = 0.15). These finding are similar but not identical to our findings and we attribute the differences to the small size of their study and only three subjects with a density greater than 30%. In our study, 48 subjects had breast density higher than 30%. Van Engeland et al. [25] compared a proprietary mammographic volumetric breast density to MRI on 22 women. MRI breast density was measured using a manual segmentation technique and the authors reported a high correlation of R^2^ = 0.94. In 26 young women, Highnam et al.[22] compared mammographic volumetric density using Volpara version 1.2.1 and found a correlation of R = 0.94 but no further analysis was offered on how the MRI density was measured or on further statistical description. Thus, including our own study, there are at least four studies with four different mammographic volumetric breast density measures that show a high correlation to volumetric breast density by MRI.

It appears that not all volumetric measures of breast density, either by MRI or by mammography, are equivalent. This lack of equivalency may or may not impact their association with breast cancer risk. For example, in fully-adjusted models of 275 breast cancer cases and 825 controls, the SXA method has been shown to have a greater association to breast cancer risk than percentage mammographic breast density [17] where the fifth to first quintile odds ratios were 4.1 for SXA breast density and 2.5 for two-dimensional breast density. To date, there have not been reports of breast cancer associations for MRI, Volpara, or Quantra measures of volumetric breast density techniques.

Our study had the following limitations: First, most MR and mammography images were not acquired at the same visit. Images acquired on the same visit could have potentially eliminated some of the observed differences. Second, we did not compare the associations to breast cancer risk across techniques. This will be done in a larger dataset now being collected.

We conclude that volumetric breast density measures of total breast volume, fibroglandular volume, and percent fibroglandular volume from screening digital mammograms calculated from the techniques used in this study are in moderate to substantial agreement with the volume measures derived from MRI. The SXA measure of density showed a higher association to MRI than Volpara or Quantra density measures. However, classification of women by volumetric density by any of the three mammographic techniques is comparable to classifications by MRI density.
